# Automated condylar seating assessment using a deep learning-based three-step approach

**DOI:** 10.1007/s00784-024-05895-w

**Published:** 2024-09-04

**Authors:** Bo Berends, Shankeeth Vinayahalingam, Frank Baan, Tabea Flügge, Thomas Maal, Stefaan Bergé, Guide de Jong, Tong Xi

**Affiliations:** 1 Department of Oral and Maxillofacial Surgery, Radboud University Medical Centre, 6500 HB, P.O. Box 9101, Nijmegen, 590 the Netherlands; 2 Radboudumc 3DLab, Radboud University Medical Centre, Nijmegen, the Netherlands; 3 Department of Oral and Maxillofacial Surgery, Charité – Universitätsmedizin Berlin, corporate member of Freie Universität Berlin and Humboldt-Universität zu Berlin, Hindenburgdamm 30, 12203 Berlin, Germany

**Keywords:** Deep learning, Condylar seating, Cone-beam computed tomography, Computer-assisted planning, Digital imaging, Orthognatic surgery

## Abstract

**Objectives:**

In orthognatic surgery, one of the primary determinants for reliable three-dimensional virtual surgery planning (3D VSP) and an accurate transfer of 3D VSP to the patient in the operation room is the condylar seating. Incorrectly seated condyles would primarily affect the accuracy of maxillary-first bimaxillary osteotomies as the maxillary repositioning is dependent on the positioning of the mandible in the cone-beam computed tomography (CBCT) scan. This study aimed to develop and validate a novel tool by utilizing a deep learning algorithm that automatically evaluates the condylar seating based on CBCT images as a proof of concept.

**Materials and methods:**

As a reference, 60 CBCT scans (120 condyles) were labeled. The automatic assessment of condylar seating included three main parts: segmentation module, ray-casting, and feed-forward neural network (FFNN). The AI-based algorithm was trained and tested using fivefold cross validation. The method’s performance was evaluated by comparing the labeled ground truth with the model predictions on the validation dataset.

**Results:**

The model achieved an accuracy of 0.80, positive predictive value of 0.61, negative predictive value of 0.9 and F1-score of 0.71. The sensitivity and specificity of the model was 0.86 and 0.78, respectively. The mean AUC over all folds was 0.87.

**Conclusion:**

The innovative integration of multi-step segmentation, ray-casting and a FFNN demonstrated to be a viable approach for automating condylar seating assessment and have obtained encouraging results.

**Clinical relevance:**

Automated condylar seating assessment using deep learning may improve orthognathic surgery, preventing errors and enhancing patient outcomes in maxillary-first bimaxillary osteotomies.

## Introduction

Orthognathic surgery is widely used to correct jaw deformities and to improve oral function and facial aesthetics. In recent years, three-dimensional virtual surgery planning (3D VSP) and computer-aided surgery (CAS) have been routinely incorporated in the daily practice to improve the efficiency of the surgical work-up and to enhance the predictability of surgical outcome [1, 2]. Numerous clinical studies have demonstrated the ability to achieve high surgical accuracy in bimaxillary orthognathic surgery by using interocclusal splints or patient-specific implants [3, 4].

One of the primary determinants for reliable VSP and an accurate transfer of VSP to the patient in the operation room is the condylar seating [5]. The 3D VSP is based on a CBCT in which the mandible is positioned in centric occlusion. An occlusal wax-bite (OWB) is commonly used to stabilize the mandible in centric occlusion, so ensuring a correct seating of the condyles during CBCT scanning. However, challenges such as suboptimal manual guided closure, incorrect placement of the OWB or shifted condylar seating during the fitting of the OWB may result in inadequately seated condyles on the CBCT [6].

Incorrectly seated condyles would primarily affect the accuracy of maxillary-first bimaxillary osteotomies as the maxillary repositioning is dependent on the positioning of the mandible in the CBCT scan [7]. Inadequate seating of the condyles can result in an erroneous position of the mandible in VSP that does not correspond to the patient’s centric occlusion in the operating room. This discrepancy could negatively influence the postoperative occlusion, facial aesthetics and postoperative stability, leading to a suboptimal outcome [8]. Thus, the condylar seating should be systematically evaluated either prior to or during VSP. The incorporation of an automated assessment tool for condylar seating could potentially warn surgeons and medical engineers in case of incorrectly seated condyles on CBCT so that adequate actions can be taken timely prior to surgery to address this problem [9].

In our previous study, a three-step deep learning approach based on 3D U-net was successfully implemented for the automated segmentation of mandibular condyles with high accuracy, consistency and efficiency [9]. The integration of this AI-segmentation tool into diagnostic software could provide the foundation for the development of an AI-based assessment tool for condylar seating. This study aimed to develop and validate a novel tool by utilizing a deep learning algorithm that automatically evaluates the condylar seating based on CBCT images as a proof of concept.

## Materials and methods

### Data

60 CBCT scans were collected from patients who underwent orthognathic surgery between 2007 and 2017 at the Department of Oral and Maxillofacial Surgery at the Radboudumc. Approval from the local Medical Ethical Committee for collecting patient data for medical research was obtained for this study (CMO Radboudumc 2020–6608). Inclusion criteria were CBCT scans on which the mandibular condyles were clearly depicted. Before inclusion, the scans were screened based on condylar seating quality to obtain a dataset in which the ratio of correctly and incorrectly seated condyles were ought to be equal. Screening took place by BB. The exclusion criteria were the presence of motion artefacts in a CBCT scan, patients under the age of 16 and a syndromic jaw disorder. CBCT scans were taken one to four weeks before surgery or up to one year after surgery. A standard CBCT scanning protocol in the “Extended Field” modus was used during scanning (field of view: 22 × 16 cm; scan time: 40 s; pixel spacing: 0.4 mm; slice thickness: 0.4 mm) at 120 kV and 5 mA pulse mode. All scans were acquired by an i-Cat CBCT scanner (i-CAT, 3D Imaging System, Imaging Sciences International Inc, Hatfield, PA, USA). During scanning, the patients were seated in natural head position (NHP) with the dentition in a centric relation. After scanning, the CBCT data were exported in DICOM format and anonymized before further analysis.

### Data annotation

The condylar seating was assessed on each CBCT based on the sagittal, axial, and coronal slices by four maxillofacial surgeons (MK, SB, TX, JL) and a 3D medical engineer (FB). The condylar seatings were labeled as correct or incorrect. All clinical annotators have 35, 30, 15 and 10 years of clinical experience, respectively. The 3D medical engineer has 6 years of experience in medical planning of orthognathic surgery. Each annotator was instructed and calibrated in the verification task. The final label assigned to each condyle (correctly or incorrectly seated) was determined using the majority rule. A condyle was considered incorrectly seated if it was not positioned in the center of the fossa and if the surgeon would contemplate modifying the 3D VSP or retaking the CBCT scan due to the condyle’s seating.

### Model

The automatic assessment of condylar seating included three main parts: segmentation module, ray-casting and feed-forward neural network (FFNN).

### Segmentation module

For the automatic segmentation of the condyles and the fossae, three different 3D U-Nets were utilized. The first 3D U-net was used to roughly segment the condyles from rescaled and horizontally split CBCT scans (low-resolution). From the centroid of the roughly segmented condyles, the location of the condyles and fossae were determined automatically in the original CBCT scan (high-resolution). The second 3D U-Net segmented the located condyles and fossae as one entity in the original CBCT scan. The third 3D U-Net was applied to distinguish between the segmented condyle and fossa. Subsequently, the segmented condyle was used to compute the centroid [9]. 

### Ray-casting

**Fig. 1:**
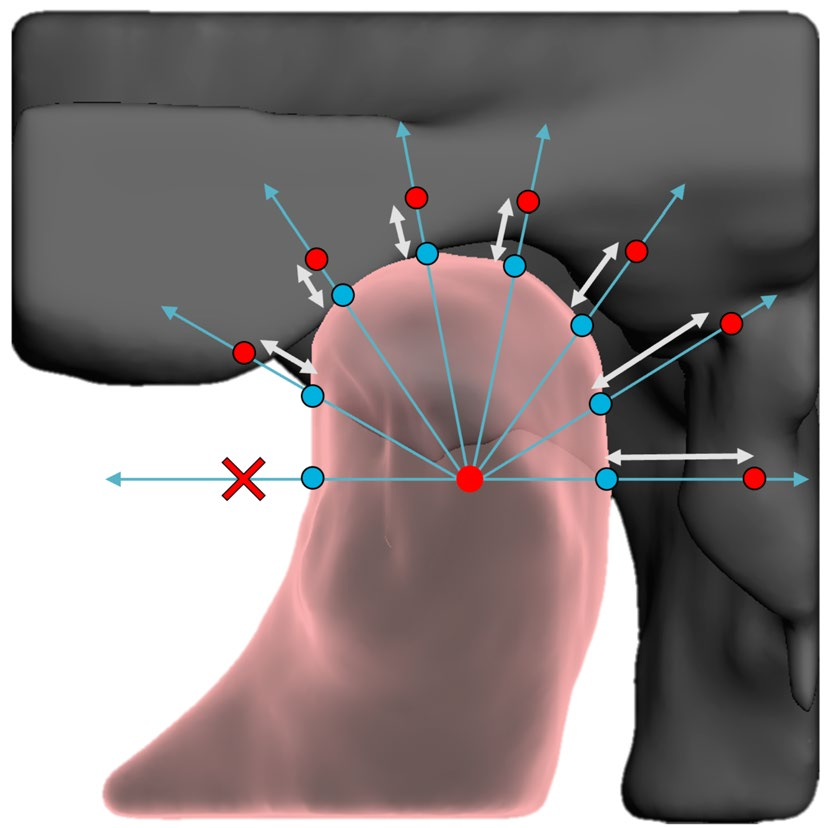
Is an illustration of the ray-casting method in which a hemi-icosphere was placed in the centroid of the condyle Rays (blue arrows) were cast from the centroid of the condyle in the direction of all 198 vertices of the hemi-icosphere. The distances (white arrows) between the intersection of the rays with the condylar surface (blue spheres) and glenoid fossa (red spheres) were computed. A cross illustrated whether a ray had no intersection with the glenoid fossa

A ray-casting algorithm was applied for each temporomandibular joint, utilizing a reference hemi-icosphere (*r* = 1 mm) consisting of 198 vertices. The center of the hemi-icosphere was placed on the location of the computed centroid and the rays were cast outward in the direction of the 198 vertices, as can be seen in Fig. 1. The orientation of the hemi-icophere was aligned with the global coordinate system of the CBCT device. Subsequently, the length between the intersection of the condyle and fossa was calculated for each ray and forwarded to the FFNN. If a ray did not intersect the glenoid fossa, thereby impeding the computation of condyle-fossa distances, a value ranging between the minimum and maximum computed ray distances were assigned to the ray.

### Feed-forward neural network

A conventional FFNN with 192, 128, 64 and 32 nodes within the hidden layers was used for the binary classification. The rectified Linear unit (ReLu) was implemented as the activation function within the hidden layers, whereas the sigmoid activation function was applied in the output layer.

Fivefold cross validation was used during training (80%) and testing (20%). Throughout the process of five-fold cross-validation, four consecutive folds were allocated for training purposes, while the fifth fold was reserved for testing. This cycle was repeated five times to ensure comprehensive evaluation. The train-test split was stratified based on the amount of correctly and incorrectly seated condyles.

The FFNN optimization used an Adam optimizer at a decaying triangular cyclic learning rate with a base learning rate of 5 × 10^− 5^, a maximum learning rate of 5 × 10^− 4^ and a step size of 2,000. Class balancing was used to compensate for an unequal distribution of correctly and incorrectly seated condyles in the datasets. Furthermore, a binary cross-entropy loss, a batch size of 32, batch normalization with a momentum of 0.8 and a dropout rate of 0.25 were applied. The models were implemented in Keras and TensorFlow on a 12 GB NVIDIA TITAN V GPU and for each fold trained for 1,000 epochs. After training, the network had a confidence value ranging between 0 and 1 as output. A prediction cut-off of 0.2 was used to distinguish if the network’s prediction meant a correct (confidence value < 0.2) or an incorrect condylar seating (confidence value ≥ 0.2).

### Data augmentation

To enhance the robustness of the network during training, several data augmentation techniques were employed to the training data. First, copies of the condyle/fossa pairs were mirrored along the midline to simulate the contralateral condyle/fossa pairs, thereby increasing the amount of data that was used for training. Subsequently, to reduce the network’s dependence on the precise position of the hemi-icosphere within the condyle during ray-casting, the hemi-icosphere was randomly translated between − 2 and + 2 millimeters in all directions for each epoch. Additionally, to mitigate the effects of head tilt during CBCT scanning, the hemi-icosphere was randomly rotated between − 10 and + 10 degrees in all directions for each epoch. This simulated slight deviations in the patient’s head orientation during scanning. These augmentation strategies ensure the network becomes more resilient to variations in the input data, improving its generalization capabilities.

### Statistical analysis

The condylar seating was assessed based on the true positives (TP), true negatives (TN), false positives (FP) and false negatives (FN) on the condyles of the validation dataset. Classification metrics are reported as follows for the test set: accuracy = $\:\frac{TP+TN}{TP+TN+FP+FN}$, precision = $\:\frac{TP}{TP+FP}$ (also known as positive predictive value), dice = $\:\frac{2TP}{2TP+FP+FN}$ (also known as the F1-score), recall = $\:\frac{TP}{TP+FN}$ (also known as sensitivity), specificity = $\:\frac{TN}{TN+FP}$ and negative predictive value = $\:\frac{TN}{TN+FN}$. Furthermore, the area-under-the-curve-receiver-operating-characteristics-curve (AUC) and confusion matrix are presented.

## Results

**Fig. 2:**
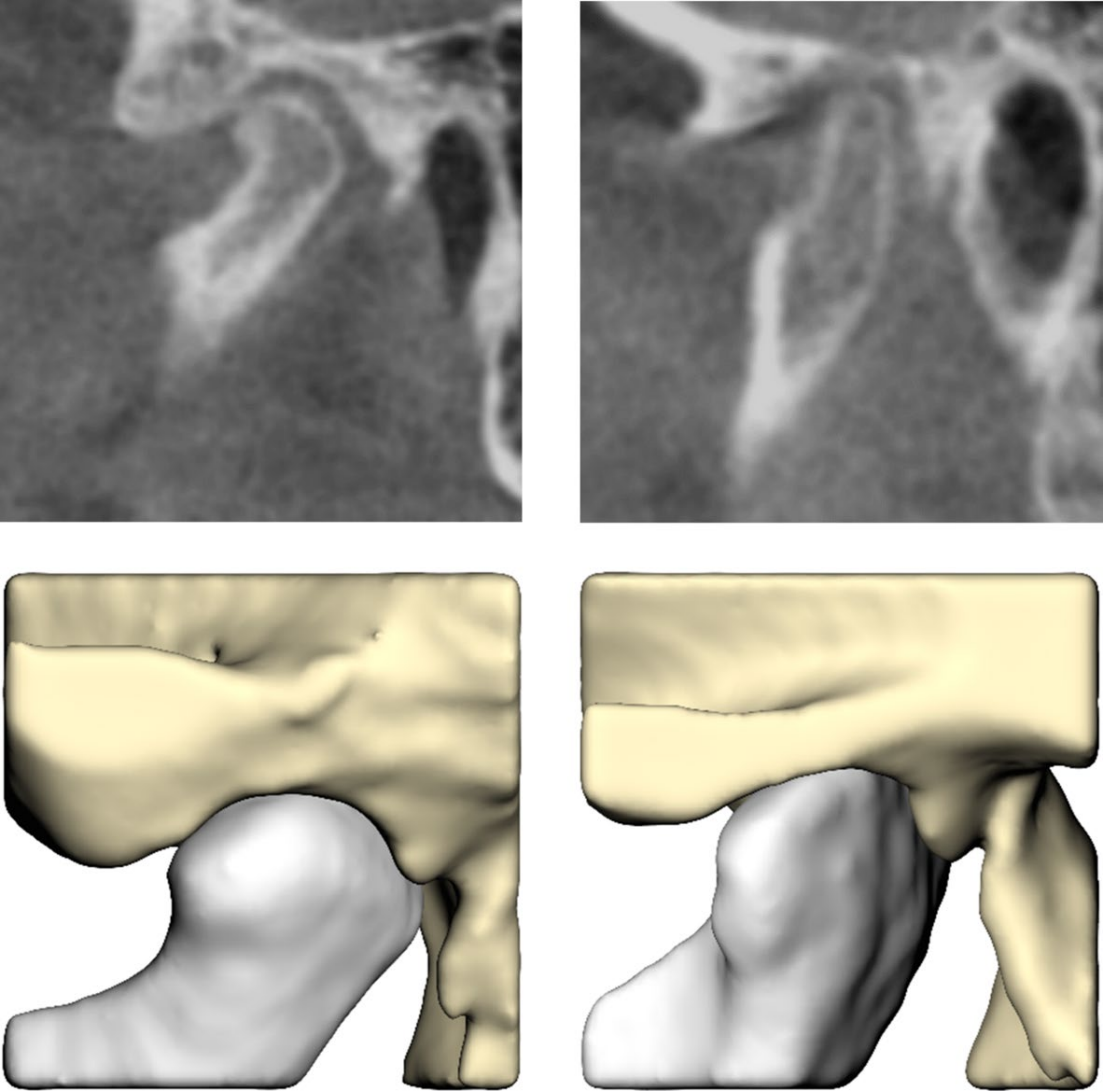
Sagittal slices (upper) and their corresponding segmentations (lower) of condyles annotated as correctly seated

**Fig. 3:**
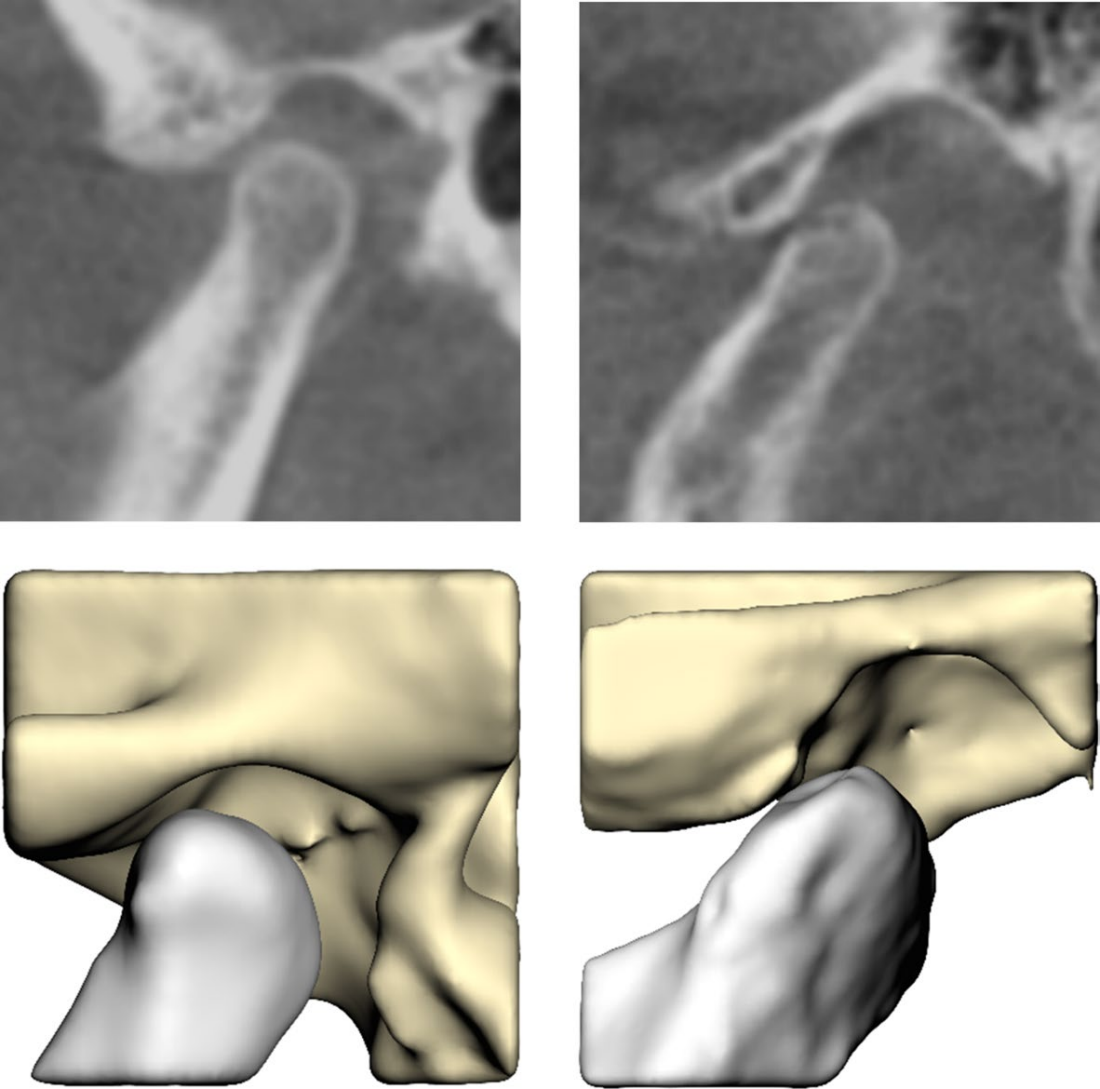
Sagittal slices (upper) and their corresponding segmentations (lower) of condyles annotated as incorrectly seated

The 60 CBCT scans that were included resulted in 120 condyle-fossa combinations that were annotated and segmented. Of the 120 condyles, 85 condyles (71%) were assessed as being correctly seated, whereas 35 condyles (29%) had a suboptimal seating. For 40 condyles (33%) the majority-rule was used to come to a consensus, whereas for 80 condyles (67%) the condylar seating was assessed unanimously (Table 1). Sagittal slices and their corresponding segmentations of condyles annotated as correctly and incorrectly seated can be found in Figs. 2 and 3, respectively. As a fivefold cross validation was used in combination with a stratified train-test split that was based on the number of correctly and incorrectly seated condyles, 17/85 unique correctly seated and 7/35 unique incorrectly seated condyles ended up in the validation dataset for each fold. Thereby, each condyle ended up once in the validation dataset.

**Table 1:** Assessment of the condylar seating by maxillofacial surgeons and medical engineers

Number of surgeons that marked the condylar seating as incorrectly	0	1	2	3	4	5
Number of condyles	63	12	10	14	4	17

**Fig. 4:**
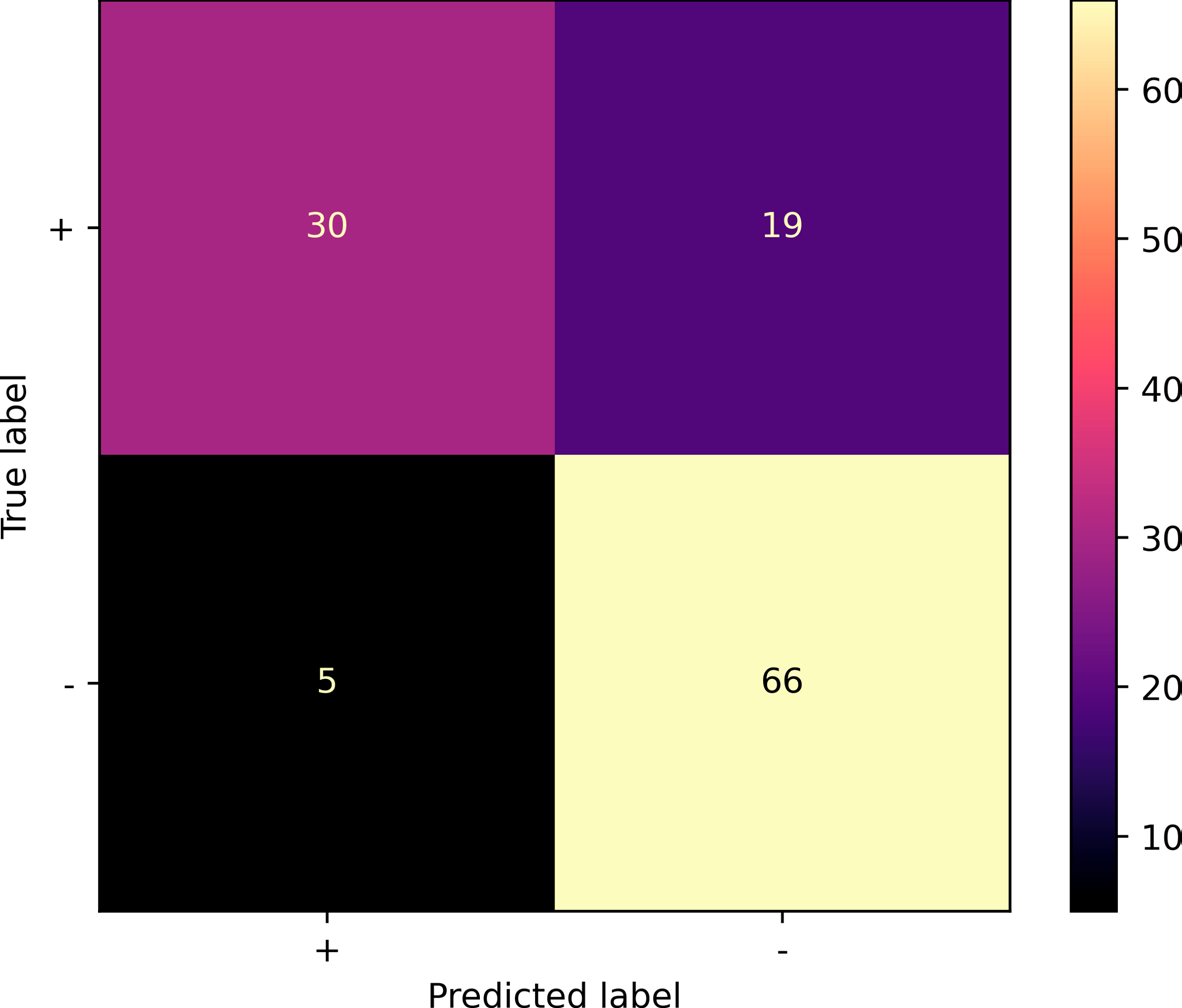
Confusion matrix illustrating the classification results of condylar seating over the 5 validation-folds. + stands for correctly seated condyles; - represents the incorrectly seated condyles

**Fig. 5:**
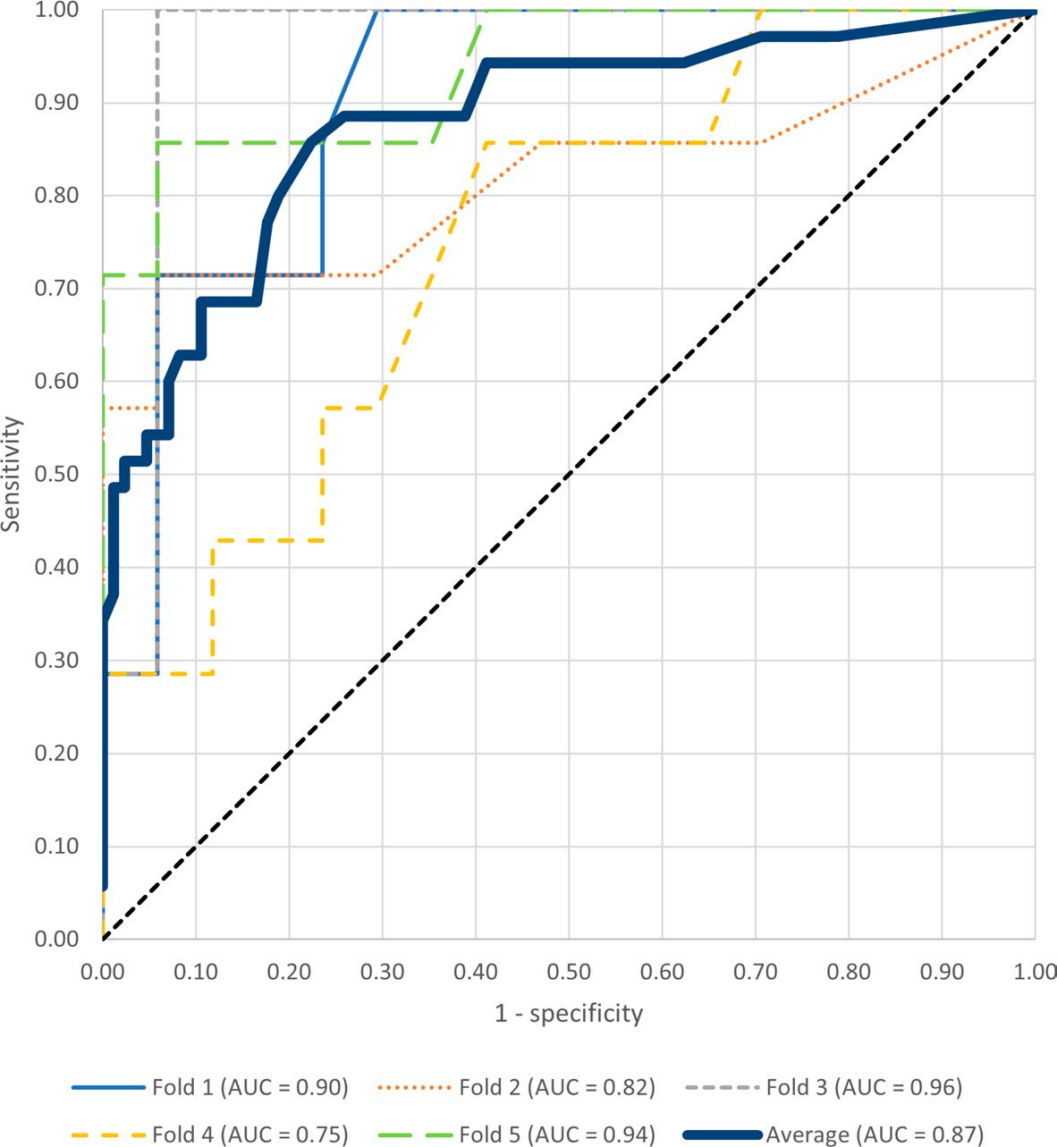
Area-under-the-curve-receiver-operating-characteristics-curve of the seating prediction of the condyles in the validation datasets

The automated workflow was able to assess the condylar seating by providing an output in terms of a well-seated or a suboptimal seated condyle. The confusion matrix in Fig. 4 illustrates the classification performance. The model achieved an accuracy of 0.80, positive predictive value of 0.61, negative predictive value of 0.93, and a F1-score of 0.71 on the validation dataset. The sensitivity and specificity of the model was 0.86 and 0.78, respectively. The mean AUC over all folds was 0.87 for the validation dataset (Figs. 5) and 0.90 for the training dataset. The model achieved an accuracy of 0.85 for the 80 condyles that were annotated unanimously, whereas this was 0.70 for the 40 condyles in which the majority rule had to be used to assign the final label.

## Discussion

The purpose of this study was to develop and validate a new tool based on deep learning that is able to automatically evaluate the condylar seating using CBCT. By adopting a workflow that was consisted of a segmentation module (3D U-Net), ray-casting and FFNN, a dichotomic outcome in terms of a well-seated or a suboptimal seated condyle could be generated from the CBCT scan that was used as an input [9]. The evaluation metrics for condylar seating assessment showed encouraging results, with an accuracy of 80% and an AUC of 0.86. The model achieved an accuracy of 80% and an AUC of 0.86. Sensitivity was high at 86%, effectively detecting incorrectly seated condyles. Specificity was 78%, indicating the model’s ability to identify the correctly seated condyles. Although the precision was 61%, which suggested a further potential to reducing false positive outcomes, the high negative predictive value of 93% underlined the reliable identification of true negative cases.

In this study, a prediction cut-off value was strategically chosen to achieve a favorable balance between sensitivity and specificity. Choosing a lower cut-off value would improve sensitivity but might also result in more false predictions. As this model is being used as assistant tool in the 3D VSP of orthognathic surgery, a higher specificity and negative predictive value are preferred. This ensures that the surgeon is alerted to re-assess the condylar seating whenever the model identifies a potential suboptimal condylar seating.

In case that the surgeon confirms the presence of a sub-optimally seated condyle, modifications to the 3D VSP of the intended bimaxillary osteotomies would be necessary. There are several clinical alternatives in this situation. One option is to use a mandible-first sequence in which the maxillary position in VSP is not determined by the incorrectly seated condyle [10]. If this approach is less desired, retaking the CBCT with a correct seating of the condyles could also be considered. Moreover, by using patient-specific implants (patient-specific plates), the maxilla can also be repositioned accurately to the desired position in VSP, eliminating the necessity to have a perfectly seated condyle within the VSP [11].

The strength of the present study was the description of a new approach that strategically integrated three distinct innovations: a multi-step segmentation employing 3D U-Nets to ensure precise differentiation between condyles and fossae, a ray-casting technique utilizing reference hemi-icospheres for meticulous 3D assessment, and a FFNN facilitating binary classification using ray-cast lengths. This cohesive methodology introduced a holistic and geometry-focused approach for automating condylar seating assessment. By combining these novel elements, the study offered a viable clinical assistance tool focused on the evaluation of condylar seating by using CBCT.

Up till today, studies have primarily focused on the automated segmentation of temporomandibular joints in CBCT images. Kim et al. introduced a modified U-net combined with target region classification to automate cortical thickness measurement [12]. They achieved an average Intersection over Union (IoU) of 0.870 for marrow bone and 0.734 for cortical bone. Le et al. employed a U-net using a 2D slice-by-slice segmentation method, attaining a commendable dice score of 0.91 for mandibular ramus and condyles [13]. Verhelst et al. used a layered 3D U-Net architecture AI model to automatically create a 3D surface of the mandible from CBCT images and obtained an IoU of 0.94 for user refined AI segmentations [14]. The three-step 3D U-Net based segmentation module of the present study obtained an IoU of 0.96 [9]. The conclusion from these studies was that the AI model could provide a time-efficient, accurate and reproducible workflow for the creation of 3D condylar models. However, the next step is to use the automatically created condylar models for clinical assessment of TMJs to support the clinician in diagnosis and clinical decision-making. No previous studies have attempted to use these enhanced segmentations of the TMJs to evaluate the condylar seating.

An advantage of the ray casting approach is its independence from the voxel size of CBCT scans, as distances are translated into millimeters. This property facilitates the incorporation of a wider range of data for training and validation, which in turn would enhance the algorithm’s generalizability.

Furthermore, the deep learning algorithm was trained using dense matrices. This approach necessitated the representation of the fossa-condyle distances for all rays cast. However, a subset of rays did not intersect the glenoid fossa, thereby impeding the computation of condyle-fossa distances. To address this issue, such rays were assigned values ranged between the minimum and maximum computed ray distances. This strategy aimed to prevent undue algorithmic emphasis on these rays during training. An alternative strategy might exclude these non-intersecting rays from the input dataset. This could be achieved by using sparse matrices for training, which could potentially further enhance the algorithm performance. Future investigations should explore the potential benefits of sparse matrices to achieve better results in the automated evaluation of condylar seating.

Even though de Jong et al. [15]. have shown that the combination of a raycasting algorithm with FFNN could successfully be used for classification tasks, other deep learning techniques could also have been explored to refine the differentiation between correctly and incorrectly seated condyles, such as deep learning networks for 3D point cloud data [16, 17] or networks that had the original volumetric CBCT data directly as input [18]. Future research should investigate the efficacy of such methods in enhancing automated condylar seating assessments.

Although the present results are promising, there are several limitations to this study. Firstly, the method lacked a true gold standard to determine whether the condyle was seated correctly or incorrectly within the fossa. This could also be seen from the considerable variation in classifications by the annotators. Clinical consensus was reached by the five annotators for only 80 out of the 120 condyles (66.7%). The model’s performance aligned with that of the annotators as the model’s performance was inferior for the 40 condyles in which no clinical consensus was reached. The absence of a gold standard may have impacted the method’s performance, as the network could have been trained on data without a clear distinction between correctly and incorrectly seated condyles. Despite surgeons routinely classifying whether the condylar seating is adequate for 3D VSP of orthognathic surgery, these results indicate that this task is not always straightforward and might be dependent on a surgeon’s experience and preference. Therefore, a method that objectively assesses the adequacy of condylar seating during 3D VSP, such as the one developed in this study, could be highly beneficial. However, using a more detailed and outcome driven classification protocol to distinct correctly from incorrectly seated condyles would be advantageous. Ideally, a protocol or dataset should be developed in which the condylar seating was correlated to the surgical accuracy that was achieved during orthognathic surgery. This would enable the network to make more clinically relevant decisions regarding the adequacy of condylar seating for 3D VSP.

Another limitation of the study was that the dataset used exhibited class imbalance, with more instances of correctly seated condyles compared to incorrectly seated condyles. This imbalance could have adversely influenced the performance of the workflow by skewing the model’s learning process. Additionally, the performance of the Feedforward Neural Network (FFNN) could be enhanced by augmenting the training dataset, as its size was relatively limited. Furthermore, the reported study is limited by its monocentric design resulting in a database consisting of only local population. The CBCTs were acquired with only one device and did not take clinical settings into account in which CBCTs may be acquired with different scanners. Furthermore, the model was strictly confined to the employed train- and test set which may limit its performance with external and more heterogenous datasets. Training of the present model with multi-centered and labelled data may be required to increase the model’s robustness and generalizability. Prospective studies with aforementioned suggestions are required prior to the implementation of this deep learning approach in the daily practice [9].

In conclusion, the innovative integration of multi-step segmentation, ray-casting and a FFNN demonstrated to be a viable approach for automating condylar seating assessment and have obtained encouraging results. The approach offers the potential to further ease 3D VSP in orthognathic surgery.

## Data Availability

The data that support the findings of this study are available from the corresponding author upon reasonable request.
